# Belongingness in Early Secondary School: Key Factors that Primary and Secondary Schools Need to Consider

**DOI:** 10.1371/journal.pone.0136053

**Published:** 2015-09-15

**Authors:** Sharmila Vaz, Marita Falkmer, Marina Ciccarelli, Anne Passmore, Richard Parsons, Melissa Black, Belinda Cuomo, Tele Tan, Torbjörn Falkmer

**Affiliations:** 1 School of Occupational Therapy and Social Work, Curtin University, Perth, Western Australia, Australia; 2 School of Education and Communication, CHILD programme, Institution of Disability Research Jönköping University, Jönköping, Sweden; 3 School of Pharmacy, Curtin University, Perth, Western Australia, Australia; 4 Department of Mechanical Engineering, Curtin University, Perth, Western Australia, Australia; 5 Rehabilitation Medicine, Department of Medicine and Health Sciences (IMH), Faculty of Health Sciences, Linköping University & Pain and Rehabilitation Centre, UHL, County Council, Linköping, Sweden; National Center of Neurology and Psychiatry, JAPAN

## Abstract

It is unknown if, and how, students redefine their sense of school belongingness after negotiating the transition to secondary school. The current study used longitudinal data from 266 students with, and without, disabilities who negotiated the transition from 52 primary schools to 152 secondary schools. The study presents the 13 most significant personal student and contextual factors associated with belongingness in the first year of secondary school. Student perception of school belongingness was found to be stable across the transition. No variability in school belongingness due to gender, disability or household-socio-economic status (SES) was noted. Primary school belongingness accounted for 22% of the variability in secondary school belongingness. Several personal student factors (competence, coping skills) and school factors (low-level classroom task-goal orientation), which influenced belongingness in primary school, continued to influence belongingness in secondary school. In secondary school, effort-goal orientation of the student and perception of their school’s tolerance to disability were each associated with perception of school belongingness. Family factors did not influence belongingness in secondary school. Findings of the current study highlight the need for primary schools to foster belongingness among their students at an early age, and transfer students’ belongingness profiles as part of the hand-over documentation. Most of the factors that influenced school belongingness before and after the transition to secondary are amenable to change.

## Introduction

The feeling of ‘belongingness’ represents an active internal experience of a strong psychological connection [1, 2]. School belongingness, or the psychological sense of school membership, is the feeling of being “personally accepted, respected, included, and supported by others in the school social environment” [3] and is an antecedent to a successful learning experience [4–6]. Students who report greater belongingness in school are less likely to engage in health-compromising behaviours [7, 8], are more likely to have better mental health functioning [4, 9, 10] and to succeed academically [4, 11, 12]. Nurturing a sense of belonging in school is positively associated with the retention of students who are at-risk of dropping out of school [8, 13–15]. Given the detrimental effects on the individual and society of prematurely leaving school [8, 16], schools and communities face the ever-growing challenge of ensuring that students continue to belong in school [5, 17]. Accordingly, identifying key factors associated with belongingness in early secondary school could support the design of more inclusive school environments.

The need to belong in school is important in early adolescence, as students explore aspects of personal identity separate from families, and rely more on friendships and non-kin relationships for support and direction [18–21]. In most Western countries, including Australia, students negotiate the transition from primary to secondary school during early adolescence. This transition involves coping with changes in school organisational structure, social hierarchies, and social role orientation [22–24]. Students move from being the oldest in primary school to the youngest in secondary school; lose the secure peer network and single (home room) teacher base; and need to adjust to new peers and expectations of multiple teachers. These changing demands can result in the loss of a student’s key role model or adult figure, at a time in life when the need for guidance and support from non-familial adults and peers is paramount [25]. It is unknown if, and how, students redefine their sense of belonging across the primary-secondary school transition. Empirical evidence on whether students’ perceptions of school belongingness change after the transition to secondary school; and whether any change is influenced by factors such as gender, disability, or household socio-economic status (SES) has been largely unexplored.

### School belongingness across the primary-secondary school transition

Explicit research on the effects of primary-secondary school transition on adolescents’ perceptions of school belongingness is limited. Inductive studies on students’ social experiences across the transition suggest most adolescents regard relationships and the social aspects of the transfer process to be more important than academic attainment [26–28]. Most students settle into secondary school better than anticipated [29–31]; with the well-adjusted group forging friendships with classmates and positive relationships with teachers and key adults. Students from socially and academically disadvantaged backgrounds, as well as those with problem behaviours and fewer friendships prior to entering secondary school seem to be disadvantaged across the transition [32]. Although gender differences in social adjustment are reported in the literature; the specifics of these difficulties are inconsistent. For example, some researchers found that females had reduced close friendships and support after transition, while males had increased school problems during the transition period (e.g. [33]). Others suggest that females negotiate the transition into secondary school easier than males (e.g. [31]). Poorer social adjustment among students with a disability has been attributed to several factors, including: poorer social skills [34]; lower social acceptance by peers and fewer friendships [35, 36]; and weaker classmate and parental support [37]. Deductive studies [38, 39] have substantiated the within-cohort variability in social adjustment among school students. Most studies conducted in the United States of America (US) [11, 40–43] suggest school belongingness reduces as students’ progress through secondary school and have credited the reductions to the ‘stage-environment misfit’ hypothesis. The hypothesis conceptualises the schooling transition as less as a consequence of adolescence, but rather of differences between primary and secondary school classroom environments.

Australian research on the effect of primary-secondary school transition on school belongingness is scant. An Australian study, conducted by Vaz et al., [44, 45] followed a cohort of students from 75 primary schools into 152 secondary schools to determine the impact of the transition on students’ academic performance, social and emotional adjustment (school belongingness, loneliness and mental health) and participatory outcomes. Using cross-sectional data from 395 students, Vaz et al., [46] outlined the 15 most significant personal-student and contextual factors that explained 66.4% (two-thirds) of the variability students’ perceptions of belongingness in the final year of primary school. Females and students with disabilities reported higher school belongingness than males and their typically developing peers, respectively. No variability in school belongingness due to household-SES was identified. The majority (41.9% out of 66.4%) of the variability in primary school belongingness was explained by personal-student attributes, such as social acceptance competence, physical appearance competence, coping skills and motivation. The remainder was accounted for by parental expectations (additional 3%), followed by school and classroom based factors (additional 13.9%) such as, classroom involvement, task-goal structure, autonomy provision, cultural pluralism, and absence of bullying. Whether the factors identified as influencing school belongingness in the final year of primary school [46] remain in place, once students transition to secondary school is yet to be determined.

## Aims and Objectives

The current study builds on the previous work by Vaz et al., [46] and uses longitudinal data from the same student cohort of students who moved from primary to secondary school to address five objectives:

- Objective 1: determine whether students’ perceptions of school belongingness changes across the primary-secondary school transition, and if so, whether gender, disability and household- SES influence the change;
- Objective 2: determine whether factors identified by Vaz et al., [46] to be associated with belongingness in primary school, continue to be associated with belongingness in secondary school;
- Objective 3: after controlling for primary school belongingness, to determine whether factors identified by Vaz et al., [46] to be associated with belongingness in primary school, maintain their influence on belongingness in secondary school;
- Objective 4: after controlling for primary school belongingness, to determine whether there are additional factors; and
- Objective 5: in the event that additional factors that influence belongingness in secondary school are identified, to develop the best-fit model of belongingness in secondary school, after accounting for primary school belongingness.

## Methods

### Study design

A prospective, longitudinal design with two data collection points at Time 1 (T1) in primary school and Time 2 (T2) in secondary school was used. Survey questionnaires were used to retrieve information. At T1, information was collected from students (with and without disabilities), a primary caregiver (parent or guardian) and class teacher. T1 data collection occurred six months prior to the transition to either middle or secondary school. At T1, data from 395 students from 75 primary schools were collected.

T2 data collection occurred six months after the transition to secondary school. Detailed information on the study design, research participants, recruitment, and data collection methods has previously been published [44, 47, 48]. Informed written consent was obtained from school principals, parents and teachers, as well as written assent from students to participate in this study. In situations where the student declined to participate, even with parental consent, they were not included. All participants were made aware that they were not obliged to participate in the study, and were free to withdraw from the study at any time without justification or prejudice. Ethics approval was obtained from Curtin University Health Research Ethics Committee in Western Australia (WA) (HR 194/2005).

### Participants

The current study presents data from the 266 students that answered both T1 and T2 questionnaires. Access to the complete data can be obtained by contacting the first author. As previously reported in a related paper [46, 47], the mean age of students at T1 was 11.89 years (SD = 0.45 years, median = 12 years), and at T2 was 12.9 years (SD = 0.57 years, median = 13 years). Girls constituted 53.4% of the sample; and 25.9% were reported by a primary caregiver to have a disability. Students were categorised into the disability subgroup if they were reported to have a disability which impacted the student’s daily functioning. To be eligible for the study, their parent/caregiver needed to confirm that they were attending a mainstream class for at least 80% of their school hours per week, with support provided as required. Thus, a broad definition was used to categorise students into the disability group. The main disabilities included asthma (18.8%), auditory disability (15.9%), attention deficit hyperactivity disorder/ attention deficit disorders (ADHD/ADD) (14.5%), learning disability (11.6%), autism spectrum disorders (10.1%), and cerebral palsy (8.7%). Based on the Australian Bureau of Statistics median income categorisation [49], the majority of the sample were from mid-range households (58.3%, n = 154), followed by high SES households (33%, n = 87); with 8.7% (n = 23) from low-SES families.

The T1 sample represented students from 52 primary schools across 77 different classes; with 47% enrolled in public (government) schools, 29% in Catholic Education schools, and 24% in independent (non-government) schools. The T2 student sample attended 152 different secondary schools. A greater number of students were seen to shift from the government system to the privatised/independent and Catholic Education systems for their secondary education. Specifically, 28.8% and 11.2% of students shifted from the government school system to the independent and Catholic Education school systems respectively, and 11.7% moved from the Catholic Education to the independent school system. Less than 6% of the sample shifted into the government system for their secondary schooling.

### Measurement tools

Table 1 provides an overview of the tools used to measure the personal-student, family and school contextual factors associated with school belongingness at T1 and T2.

**Table 1:** Overview of key moderators, personal, and contextual factors (family and school context) considered for inclusion in the school belongingness model [46].

	Factor	Instrument/ main source	Purpose	Rater	No of items or domains and meaning of total score	Psychometric properties (if needed—addition references to substantiate psychometrics if available)
**Covariates**	**Age**	Drawn from the Indicators of Social and Family Functioning Instrument Version-1 (ISAFF) [50] and Australian Bureau of Statistics surveys	Demographic profile of the sample to match the data to normative data	Parent/ Guardian	6-items	Instrument Version-1 (ISAFF) [50] and Australian Bureau of Statistics surveys
	**Gender**	Boy/Girl/Other				
	**Presence/ absence of disability and type of disability**	Yes/no for presence of disability and open ended question to detail primary diagnosis				
**Student personal factors**	**Perceived Competence**	Self-Perception Profile for Adolescents [51]. Domains: academic competence; athletic competence; peer acceptance competence, physical appearance competence	Measures student perceived competence in various domains of functioning.	Student	5-domains Higher score = higher competence	Cronbach’s α ranges from .78 to.90 in populations of students with learning disability and behavioural disorders [51]. Considerate convergent, discriminant, and construct validity substantiated in equivalent US and Australian samples [52–54]. Discriminant validity among secondary school typically developing students, students with learning disability and behavioural disorders has been substantiated previously [55].
**Student personal factors**	**Coping skills**	Short form of the Adolescent Coping Scale (ACS) [56]. 3 coping styles: non-productive, problem solving, and reference to others.	Measures the usage and helpfulness of coping strategies in general and specific situations.	Student	3-coping styles: higher score = better coping style.	Cronbach’s α ranges from .50 (reference to others) to .66 (non-productive coping). Test-retest reliabilities range from .44 to .84 (Mean *r* = .69) [56]. Validated in Australian samples [56].
	**Motivational orientation for schooling**	Inventory of School Motivation (ISM) [57, 58]. Domains: Task goals: (Mastery) task and effort motivation, Ego goals (Performance): competition and social-power motivation, Social solidarity goals: affiliation and social concern motivation, Extrinsic goals praise and token reward.	Assesses information on the goals students adopt for schooling	Student	8-domains Higher score = higher related motivation	Cronbach’s α ranges from .53 to.81. Adequate content, construct validity and test-reliability substantiated in cross-cultural studies [58–62]
	**Expectations for schooling**	Personal expectations. Perception of teachers & parent/guardian expectations of schooling [63].	Assesses student’s expectations for schooling and their perception of their parents’ and teacher’s expectation.	Student	3-items	Cronbach’s α is .91. [63].
	**Mental health functioning**	Strength and Difficulties Questionnaire (SDQ) [12, 64] Domains: emotional, conduct problems, hyperactivity/inattention, and peer relationship	Brief screener of children and adolescents’ behaviours, emotions and relationships.	Parent/ Guardian	Overall mental health functioning score. Higher score = worse functioning (pro-social skills not included in total score)	Cronbach’s α ranges from .70-.80 [65]. Adequate discriminate and predictive validity [12, 64] Widely used in clinical populations [66] and with adolescents with intellectual disability [67, 68].
**Contextual factor: Family factors**	**Family demographics**	**Background**: Structure, family income, time spent in paid employment, parents’ educational background.	Obtains information about the family’s demographic factors	Parent/ Guardian	6-items	Adapted from [49, 50] [69] (ANZSCO) [70].
	**Perceived social support from one’s family**	Multidimensional scale of perceived social support (MSPSS) [71, 72]	Measures subjective perceptions of social support adequacy from the family	Student	1-domain. Higher score = higher support	Cronbach’s α for the total scale is .91. Subscale α = .90 to .95. Test-retest reliability coefficient of .85. Adequate factorial & concurrent validity have been documented [71, 72].
	**Family functioning**	Overall general functioning subscale of the McMaster family assessment device (FAD) [73, 74]	Measures the perception of “how the family unit works together on essential tasks”	Parent/ Guardian	1-domain. Higher score = worse functioning	Cronbach’s α for the total scale .86. 1- week, test-retest reliability = .71 Split-half coefficient = .83Good construct validity [73, 74]
	**Parental expectations of schooling for child**	Expectation of schooling [63]	Rates parental expectations for their child’s future success. Options ranged from primary level qualifications through to post-graduate degrees	Parent/ Guardian	1- item	Developed by researcher [63]
	**Parental involvement in education**	Multidimensional assessment of family involvement [75]. Domains: Home-School Communication, Home-Based Involvement, School-Based Involvement	Assesses parental involvement in their child’s education	Parent/ Guardian	3-domains Higher score = greater parent involvement	Cronbach’s α range from .84 to.91. Validity reported to be adequate [75].
**Contextual factor: School and classroom factors**	**School climate and adequacy of resources**	Type of school, services offered by school to address child’s needs. Information on the school sector, post code, number of students enrolled in each school, and organisational structure at each school was obtained from Department of Education and Training, WA records.	Obtain demographic details of the school	Parent	5- items	Developed by researcher [76, 77]. Cronbach’s α is .92.
	**Student’s perception of the classroom environment**	The Middle School Classroom Environment Indicator (MSCEI) [78] Subscales: Student cohesiveness, Ease, Autonomy, Task-Orientation, and Involvement subscales Single items on bullying and cultural/disability tolerance [79–82]	Measures students’ perception of the psychosocial features of the classroom environment. The scale is drawn from works of contemporary classroom environment research and the growing body of knowledge on middle schooling [17, 83, 84]	Student	7-domains. Higher score = better classroom environment	Cronbach’s α ranges = .63 to.81. Overall factor structure, discriminate validity, and alpha reliability of MSCEI are robust [79–82].
	**Parents’ perceptions of general invitations for involvement offered by their child’s school**	Parent Involvement Scale [85]	Measures parents’ perceptions of general invitations for involvement offered by their child’s school	Parent/ Guardian	1-domain. Higher score = higher involvement	Cronbach’s α = .78 and construct validity of this measure has been confirmed factor analysis [85].
**Outcome: School belongingness**	**School belongingness**	Psychological Sense of School Membership (PSSM) Goodenew [3, 86], Overall total score on 18-items (with a five-point response format)	To measure the degree to which a student feels accepted and included within the school	Student	1-domain. Higher score = greater belongingness	Cronbach’s α = .80. Test-retest reliability = 0.78 (4-week interval) [87] and .56 and .60 for boys and girls (12-month interval) [88]. The total PSSM scores correlate positively with school success [3, 86], lower levels of depression [88], and lower levels of anxiety [17]. PSSM has been shown to discriminate between groups of students predicted to be different in terms of their sense of belonging in school [3].

## Data Analyses

Data were analysed using the Statistical Package for the Social Sciences (SPSS Version 20) and Statistical Analysis System (SAS Version 9.2) software. Descriptive statistics were conducted to summarise the characteristics of the study sample. Chi-square tests of independence, paired sample *t-*tests and Kappa statistics were performed to identify significant changes in the categorical, continuous and binary/nominal scaled factors identified by Vaz et al., [46] across the transition.

Within the secondary school system in WA, students move between different classrooms in order to attend a series of specialist-taught classes. Students may therefore have different classmates for different subjects. For the purpose of analyses, students who attended a particular secondary school were treated as a cluster. To determine the effect of school clusters on school belongingness scores, the school level Intra Class Correlation Coefficients (ICC) was obtained, after adjustment for the demographic data of each student, i.e., gender, disability, and household-SES. Using Hierarchical Linear Modelling analyses, the ICC for the secondary school belongingness score was 5%, after adjustment for gender, disability, and household-SES. This suggests that school clusters had a very small effect on the relationship between students’ demographic factors and their secondary school belongingness scores. Hence, analyses were carried out at the level of the individual student. Data relating to the study’s objectives were analysed as described below:

### Objective 1

Paired sample *t-*test and regression analyses were conducted to determine whether students’ perceptions of school belongingness significantly changed across the T1-T2 transition and whether personal student factors (i.e., gender, disability and household-SES) and their interactions were associated with any change in belongingness over time.

### Objectives 2, 3 and 5

Careful screening of data and key assumptions of multiple regression, which include normality, linearity, homoscedasticity of residuals, absence of multicollinearity, independence of errors, and absence of outliers in dependent and independent variables were tested prior to undertaking regression analyses. A hierarchical model building process as outlined by Vaz et al., was followed [46]. This involved a 3-step logic process.

**Step 1:** Covariates of gender, disability, and household-SES and their interactions were added in step 1. Interaction terms were dropped from the model if they were found to be insignificant.

**Step 2:** Covariates + Identification of student personal and contextual factors added in each block: The covariates were added in Step 1 and stepwise backwards elimination was undertaken to identify the significant factors (*p* < .05) within personal student, family, and school contexts that were associated with school belongingness.

**Step 3:** Rating explanatory power of independent variables: the explanatory power of factors in blocks was assessed on the basis of how much each factor block added to the prediction of school belongingness, over and above that accounted for by the preceding block [89].

The order of entry of blocks into the regression models was as follows: Block 1: Covariates (gender, disability, and SES); Block 2: student personal factors; Block 3: family factors and Block 4: school factors.

### Objective 4

Linear regression models were run to identify additional factors associated with secondary school (T2) belongingness, not identified in Objectives 2 and 3.

## Results

### Impact of student attrition on their school belongingness scores

An attrition rate of 33% resulted in a T2 sample of 266 students and their parents from 152 secondary schools. Paired sample *t*-tests and chi-square analyses demonstrated that the participants who continued to be involved in the study at T2 did not differ in profile from those who discontinued involvement (based on gender, health status, SES-level, and school belongingness scores). This similarity in profile between responders and non-responders at T2 suggests that conclusions based on these responders should be a fair representation of all initial T1 participants.

### Objective 1: Changes in school belongingness scores and key predictors across the transition

Paired sample *t-*tests revealed that the overall mean belongingness score of the sample was stable across the transition from primary school to secondary school (T1 *M* (SD) = 3.90 (0.72), T2 *M* (SD) = 3.83 (.68); *p* = .188). Regression analyses revealed no within-group changes in school belongingness due to gender, disability or household-SES (*p* > .05).

There were no significant changes in student perceived social acceptance (*p* = .320), physical appearance competence (*p* = .270), or the frequency of using social affiliation goals for schooling (*p* = .891). On average, in secondary school students used fewer effort-goal motivational orientations [*t*_(249)_ = -2.35, *p =* .019] and fewer problem-solving coping strategies [*t*_(249)_ = -2.15, *p =* .032]. The use of non-productive coping strategies was stable across the transition period (*p* = .615).

Kappa statistics suggested moderate to high stability of family demographics over time (Kappa coefficient range = .60 - .89). A general shift from the public/government education system to private independent and Catholic Education systems was observed (Kappa coefficient = .64). At T2, students reported reductions in classroom task-goal structure (*p* < .001) and tolerance to cultural diversity (*p =* .023), and a trend for reduced bullying in school (Kappa coefficient = .26).

### Objective 2

As shown in Table 2, primary school (T1) factors explained 29.5% of the variability in secondary school (T2) belongingness. Vaz et al., [46] showed that variables at T1 explained 66.4% of the variance in belongingness at T1. This means that a number of factors other than those found at T1 must be related to belongingness at T2. Five T1 factors (two student and three contextual factors) continued to be associated with belongingness at T2.

**Table 2:** Objective 2: Regression of Secondary School Belongingness (T2) on variables associated with Primary School Belongingness (T1).

Model	Factors	Unstandardized Coefficients		Standardized Coefficients	t	p	95% Confidence Interval for B	
		B	Std. Error	Beta			Lower Bound	Upper Bound
**Step 1: Covariates**	(Constant)	3.80	.07		51.34	< .001	3.65	3.95
	T1 Girls	.03	.08	.02	.41	.678	-.13	.19
	T1 Disability	.02	.09	.01	.24	.808	-.16	.21
	T1 Low-Q SES household	-.28	.14	-.11	-1.89	.059	-.57	.01
	T1 High-Q SES household	.10	.09	.07	1.11	.264	-.07	.28
	R = .154, R^2^ = .024 adjusted R^2^ = .009							
	*F*_[4, 263]_ = 1.590, *p =* .177							
**Step2: Covariates + Student personal factors**	(Constant)	3.46	.35		9.86	< .001	2.77	4.15
	T1 Girls	.09	.07	.06	1.20	.228	-.05	.24
	T1 Disability	.13	.09	.08	1.46	.145	-.04	.31
	T1 Low-Q SES household	-.22	.13	-.09	-1.64	.101	-.49	.04
	T1 High-Q SES household	-.01	.08	-.01	-.21	.828	-.18	.14
	T1 Social acceptance competence	.04	.06	.04	.64	.523	-.09	.17
	T1 Physical appearance competence	.11	.06	.11	1.85	.065	-.00	.22
	T1 Low-Q cope solve the problem	-.28	.09	-.18	-2.87	.004	-.47	-.08
	T1 Non-productive coping	-.01	.01	-.20	-3.37	.001	-.01	-.01
	T1 Affiliation motivation	.12	.04	.17	2.93	.004	.04	.20
	F _[9, 258]_ = 7.170, *p* < .001							
	R^2^ Change = .176, R = .447, R^2^ = .200, adjusted R^2^ = .172, F statistic for change in R^2^ = 11.383, *p* < .001							
**Step3: Covariates + Student personal factors + family factors**	(Constant)	3.38	.34		9.72	< .001	2.70	4.07
	T1 Girls	.06	.07	.04	.80	.425	-.09	.21
	T1 Disability	.17	.09	.11	1.90	.058	-.001	.35
	T1 Low-Q SES household	-.20	.13	-.08	-1.55	.121	-.47	.05
	T1 High-Q SES household	-.09	.08	-.06	-1.09	.274	-.26	.07
	T1 Social acceptance competence	.02	.06	.02	.29	.766	-.11	.15
	T1 Physical appearance competence	.11	.05	.11	1.89	.059	-.004	.22
	T1 Low-Q cope solve the problem	-.23	.09	-.15	-2.50	.013	-.42	-.05
	T1 Non-productive coping	-.01	.00	-.17	-3.00	.003	-.01	-.003
	T1 Affiliation motivation	.12	.04	.17	3.12	.002	.04	.20
	T1Trade Vs University expectations for child	.23	.08	.16	2.74	.006	.06	.39
	T1 Low-Q school-based involvement by parent	-.23	.08	-.15	-2.66	.008	-.41	-.06
	F _[11, 256]_ = 7.561, *p* < .001							
	R^2^ Change = .045, R = .495, R^2^ = .245, adjusted R^2^ = .213, F statistic for change in R^2^ = 7.656, *p* < .001							
**Step3: Covariates + Student personal factors + family factors + school and classroom factors**	(Constant)	2.38	.44		5.32	< .001	1.50	3.27
	T1 Girls	.05	.07	.03	.67	.501	-.09	.20
	T1 Disability	.11	.09	.07	1.27	.205	-.06	.29
	T1 Low-Q SES household	-.20	.13	-.08	-1.55	.120	-.46	.05
	T1 High-Q SES household	-.09	.08	-.06	-1.09	.277	-.26	.07
	T1 Social acceptance competence	.01	.06	.01	.22	.822	-.11	.14
	T1 Physical appearance competence	.07	.05	.08	1.33	.182	-.03	.19
	T1 Low-Q cope solve the problem	-.10	.10	-.06	-.98	.325	-.30	.10
	T1 Non-productive coping	-.01	.00	-.15	-2.52	.012	-.01	-.00
	T1 Affiliation motivation	.10	.04	.14	2.48	.014	.02	.18
	T1Trade Vs University expectations for child	.19	.08	.13	2.25	.025	.02	.35
	T1 Low-Q school-based involvement by parent	-.23	.09	-.14	-2.57	.011	-.41	-.05
	T1 classroom involvement	.09	.07	.09	1.32	.186	-.04	.23
	T1 Low-Q task goal orientation	-.02	.10	-.01	-.23	.813	-.22	.17
	T1 Autonomy provision	.12	.06	.14	2.15	.032	.01	.24
	T1 Low-Q parental invitation for involvement	.01	.08	.00	.10	.915	-.15	.17
	T1 Cultural pluralism	.07	.05	.08	1.28	.199	-.04	.19
	T1 Disagree Vs Agree to being bullied.	.03	.08	.02	.39	.691	-.13	.19
	F _[17, 250]_ = 6.158, *p* < .001							
	R^2^ Change = .050, R = .539, R^2^ = .295, adjusted R^2^ = .247, F statistic for change in R^2^ = 2.951, *p* < .001							

#### Covariates

No variability in belongingness at T2 due to gender, disability or household-SES, was found, as well as no interactions between the covariates.

#### Student personal factors

Students who frequently resorted to non-productive coping strategies at T1 (Beta = -.15, *p =* .012) continued to report lower belongingness at T2. The pursuit of higher social affiliation goal orientations at T1 was beneficial to T2 belongingness (Beta = .14, *p =* .014).

#### Family factors

Students whose parents reported less-than-average (low-quartile) school-based involvement at T1 continued to perceive low school belongingness at T2 (Beta = -.14, *p =* .011). Students with parents who had high scholastic expectations for them in primary school (T1) were more likely to belong in secondary school (T2) (Beta = .13, *p =* .025).

#### School and classroom factors

Belonging to a classroom that provided high-level autonomy for students at T1 was beneficial to those students’ perceived school belongingness at T2 (Beta = .14, *p =* .032).

### Objective 3

**Fig 1:**
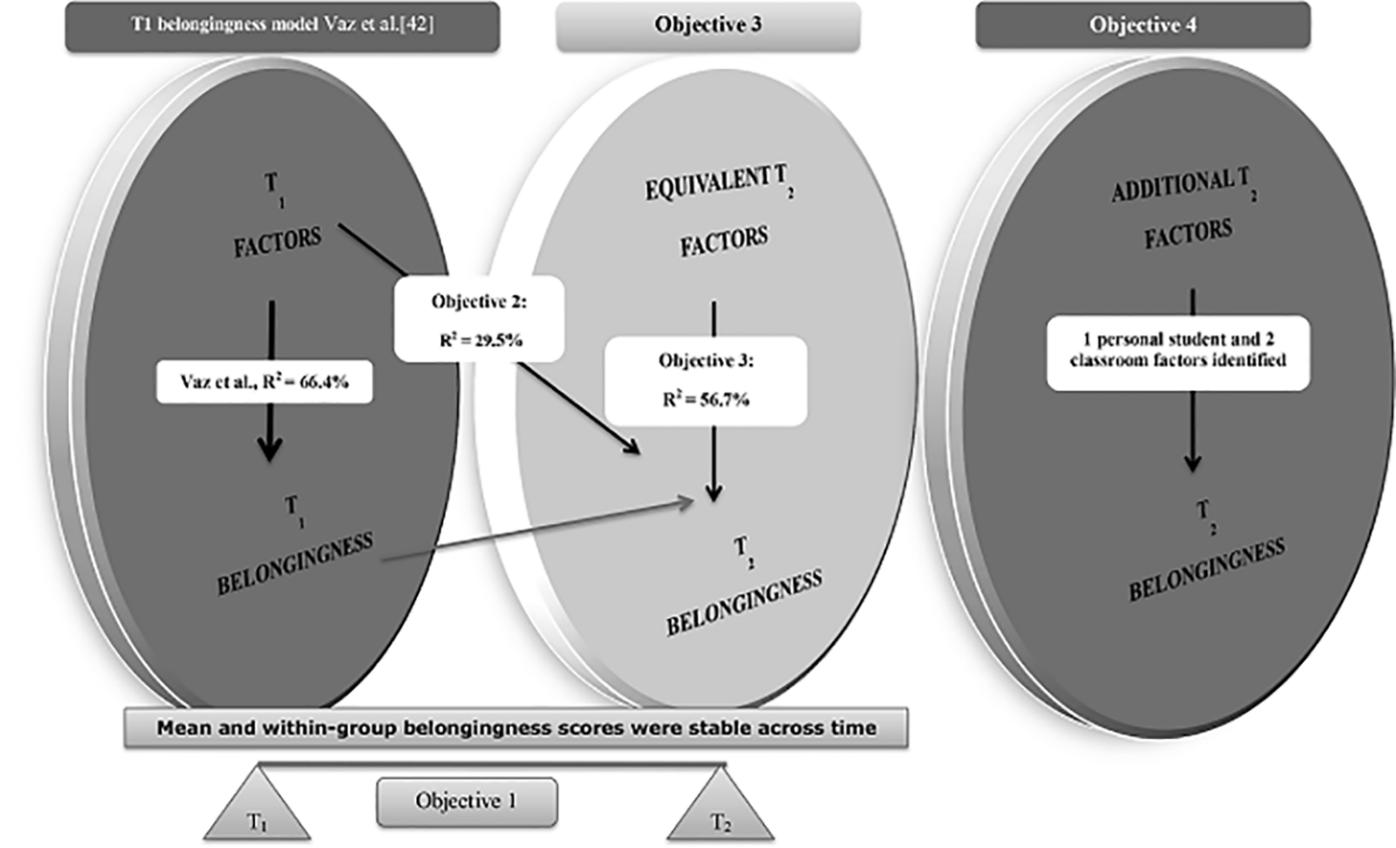
Models of Belongingness in School across the Primary-Secondary School transition.

After controlling for primary school (T1) belongingness, and using equivalent secondary school (T2) factors, the final hierarchical linear regression model explained 56.7% of variance in secondary school belongingness [*F*_(18, 232)_ = 16.851, *p <* .001]. The predictive power of the T1 model reduced from 66.4% when tested at T2 (Table 3, Fig 1).

**Table 3:** Regression of Secondary School Belongingness (T1) on Primary School Belongingness (T1), demographic and other variables associated with T1 belongingness, and; evaluated at T2.

Model	Factors	Unstandardized Coefficients		Standardized Coefficients	t	p.	95% Confidence Interval for B	
		B	Std. Error	Beta			Lower Bound	Upper Bound
**Step 1:T1 School belongingness scores**	(Constant)	2.19	.19		11.07	< .001	1.80	2.59
	T1 School belongingness	.42	.05	.47	8.45	< .001	.32	.52
	R = .472, R^2^ = .223 adjusted R^2^ = .220, *p <* .001							
	F _[1, 249]_ = 71.439, *p* < .001							
**Step 2: T1 School belongingness scores + Covariates**	(Constant)	2.24	.20		10.90	< .001	1.83	2.65
	T1 School belongingness	.41	.05	.46	8.11	< .001	.31	.51
	T1 Girls	.01	.07	.01	.25	.800	-.12	.15
	T1 Disability	.03	.08	.02	.43	.667	-.12	.19
	T1 Low-Q SES household	-.28	.12	-.12	-2.21	.028	-.53	-.03
	T1 High-Q SES household	-.01	.07	-.01	-.08	.935	-.16	.14
	R^2^ Change = .016, R = .489, R^2^ = .239, adjusted R^2^ = .223, F statistic for change in R^2^ = 1.287, *p* > 0.05							
	*F*_[5, 245]_ *=* 15.383, *p <* .001							
**Step 3: T1 School belongingness scores + Covariates+ Student personal factors**	(Constant)	2.11	.33		6.32	< .001	1.45	2.77
	T1 School belongingness	.18	.04	.20	3.67	< .001	.08	.27
	T1 Girls	.03	.06	.02	.58	.557	-.08	.15
	T1 Disability	.13	.07	.09	1.85	.064	-.00	.27
	T1 Low-Q SES household	-.20	.10	-.09	-1.90	.058	-.42	.01
	T1 High-Q SES household	-.05	.06	-.03	-.75	.451	-.18	.08
	T2 Social acceptance competence	.21	.06	.19	3.40	.001	.09	.33
	T2 Physical appearance competence	.19	.05	.21	3.92	< .001	.09	.29
	T2 Low-Q cope solve the problem	-.25	.07	-.18	-3.47	.001	-.40	-.11
	T2 Non-productive coping	-.02	.01	-.17	-3.30	.001	-.03	-.01
	T2 Affiliation motivation	.08	.03	.12	2.43	.015	.01	.15
	R^2^ Change = .235, R = .689, R^2^ = .474, adjusted R^2^ = .452, F statistic for change in R^2^ = 21.461, *p* < .001.							
	F _[10, 240]_ = 21.634, p < .001							
**Step 4: T1 School belongingness scores + Covariates+ Student personal factors**+ family factors	(Constant)	2.15	.34		6.29	< .001	1.48	2.83
	T1 School belongingness	.17	.05	.19	3.45	.001	.07	.26
	T1 Girls	.02	.06	.02	.41	.680	-.09	.14
	T1 Disability	.14	.07	.09	1.96	.051	< .001	.28
	T1 Low-Q SES household	-.20	.11	-.09	-1.90	.058	-.42	.01
	T1 High-Q SES household	-.07	.07	-.05	-1.07	.283	-.21	.06
	T2 Social acceptance competence	.21	.06	.19	3.38	.001	.08	.33
	T2 Physical appearance competence	.19	.05	.21	3.86	< .001	.09	.29
	T2 Low-Q cope solve the problem	-.26	.07	-.18	-3.53	< .001	-.41	-.11
	T2 Non-productive coping	-.01	.00	-.17	-3.16	.002	-.03	-.01
	T2 Affiliation motivation	.08	.03	.12	2.47	.014	.01	.16
	T2Trade Vs University expectations for child	.04	.06	.03	.64	.518	-.09	.18
	T2 Low-Q school-based involvement by parent	-.08	.06	-.06	-1.28	.199	-.20	.04
	R^2^ Change = .004, R = .692, R^2^ = .478, adjusted R^2^ = .452, F statistic for change in R^2^ = .998, *p* > .05							
	*F*_[12, 238]_ = 18.194, *p <* .001							
**Step 5: T1 School belongingness scores + Covariates+ Student personal factors**+ family factors+ school and classroom factors	(Constant)	1.79	.39		4.59	< .001	1.02	2.56
	T1 School belongingness	.09	.04	.10	1.86	.063	-.00	.18
	T1 Girls	.03	.05	.02	.65	.517	-.07	.15
	T1 Disability	.06	.06	.04	1.01	.312	-.06	.20
	T1 Low-Q SES household	-.12	.10	-.05	-1.22	.223	-.32	.07
	T1 High-Q SES household	-.12	.06	-.08	-1.83	.068	-.24	.01
	T2 Social acceptance competence	.18	.06	.16	3.04	.003	.06	.30
	T2 Physical appearance competence	.14	.04	.16	3.10	.002	.05	.24
	T2 Low-Q cope solve the problem	-.19	.07	-.13	-2.72	.007	-.32	-.05
	T2 Non-productive coping	-.01	.00	-.15	-3.12	.002	-.02	-.01
	T2 Affiliation motivation	.05	.03	.07	1.66	.097	-.01	.12
	T2Trade Vs University expectations for child	.01	.06	.01	.18	.854	-.11	.13
	T2 Low-Q school-based involvement by parent	-.08	.06	-.06	-1.34	.180	-.19	.03
	T2 Class involvement	.06	.06	.06	1.15	.251	-.04	.18
	T2 Low-Q task goal orientation	-.20	.07	-.15	-2.82	.005	-.34	-.06
	T2 Autonomy provision	.02	.01	.13	2.41	.017	.01	.05
	T2 Low-Q parental invitation for involvement	-.01	.06	-.01	-.27	.783	-.13	.10
	T2 Cultural pluralism	.05	.02	.11	2.16	.031	.00	.10
	R^2^ Change = .088, R = .753, R^2^ = .567, adjusted R^2^ = .533, F statistic for change in R^2^ = 7.867, *p* < .001							
	*F*_[18, 232]_ = 16.851, *p* < .001							

#### Covariates

There was no association between secondary school belongingness and gender or disability. Relative to their primary school belongingness scores, all students experienced a decline in belongingness, making sub-group differences insignificant.

#### Student personal factors

Four personal student attributes continued to be associated with school belongingness in the transition from primary school (T1) to secondary school (T2): social acceptance (Beta = .16, *p =* .003); physical appearance competence (Beta = .16, *p =* .002); low-levels of problem-solving coping skills relative to the average problem-solving group (Beta = -.13, *p =* .007), and frequent use of non-productive coping strategies (such as worrying, ignoring the problem at hand, and self-blame) (Beta = -.15, *p =* .002). In secondary school, the pursuit of social affiliation goals for schooling was no longer associated with school belongingness.

#### School and classroom factors

Belonging to culturally pluralistic classrooms that encouraged students to mix with each other and participate in important school activities continued to be associated with higher belongingness (Beta = .11 *p =* .031). Secondary school students who perceived their classrooms to be low on task- goal orientations (Beta = -.15, *p =* .005) were less likely to belong. Belonging to autonomy-granting classrooms was positively associated with belongingness in secondary school (Beta = .13, *p =* .017). In secondary school, classroom involvement and reports of being bullied in school were not associated with belongingness.

#### Family factors

Family factors did not explain any additional variance in secondary school belongingness, beyond that accounted for by preceding demographic factors and personal student, school and classroom attributes.

### Objective 4

A series of stepwise linear regression analyses identified that students who frequently adopted effort-goal motivations were more likely to perceive belongingness in secondary school. Students who believed their classrooms had high-level task-goal orientations (*p <* .001), and increased tolerance to disability (*p <* .001) were more likely to belong in secondary school.

### Objective 5

**Fig 2:**
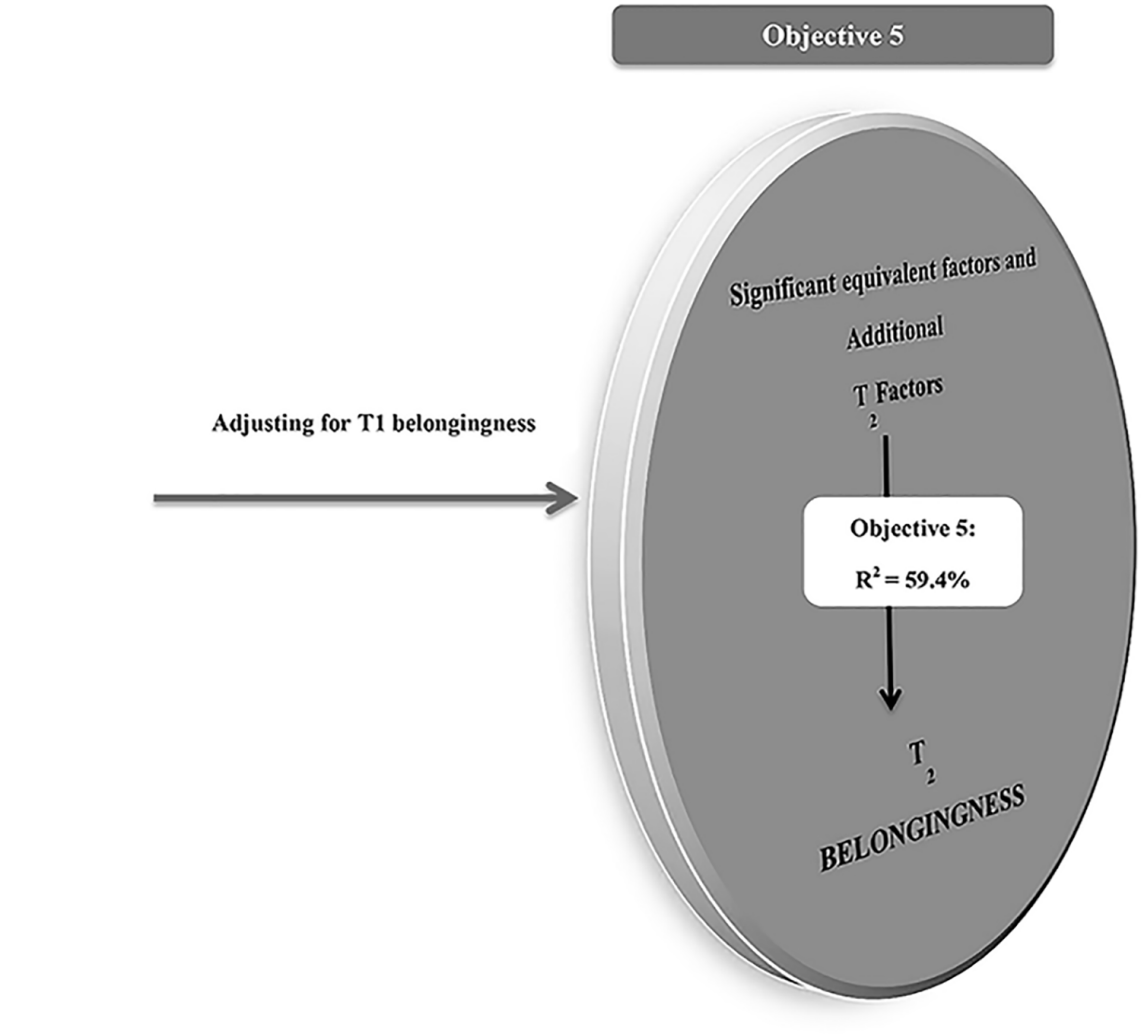
Model of Belongingness in Secondary School (T1), after accounting for Primary School (T1) Belongingness.

Fig 2 and Table 4 shows that the final hierarchical regression model explained 59.4% of variability in secondary school belongingness, (*F*_(13, 248)_ = 27.06, *p* < .001). The key contributors of secondary school belongingness were:

**Table 4:** Regression of Secondary School Belongingness (T2) on Primary School Belongingness (T1), demographic and other significant variables when evaluated at T2 and additional factors unique to T2 belongingness.

****Model****	****Factors****	****Unstandardized Coefficients****		****Standardized Coefficients****	****t****	****p****	****95% Confidence Interval for B****	
		****B****	****Std. Error****	****Beta****			****Lower Bound****	****Upper Bound****
**Step 1: T1 School belongingness scores**	(Constant)	2.1	.19		10.97	< .001	1.78	2.56
	T1 School belongingness	.43	.05	.47	8.61	< .001	.33	.52
	R = .471, R^2^ = .222 adjusted R^2^ = .219, *p <* .001							
	F _[1, 261]_ = 74.134, *p* < .001							
**Step 2: T1 School belongingness scores + Covariates**	(Constant)	2.21	.21		10.78	< .001	1.80	2.61
	T1 School belongingness	.42	.05	.46	8.24	< .001	.32	.52
	T1 Girls	.01	.07	.01	.10	.919	-.13	.14
	T1 Disability	.04	.08	.03	.56	.572	-.11	.20
	T1 Low-Q SES household	-.26	.12	-.11	-2.09	.037	-.51	-.01
	T1 High-Q SES household	.01	.07	.01	.04	.965	-.14	.15
	R^2^ Change = .014, R = .486, R^2^ = .236, adjusted R^2^ = .221, F change for R^2^ = 1.194, *p* > .05							
	*F*_[5, 256]_ = 15.826, *p <* .001							
**Step 3: T1 School belongingness scores + Covariates+ Student personal factors** + **T2 unique student personal factors**	(Constant)	1.33	.33		4.02	< .001	.68	1.99
	T1 School belongingness	.15	.04	.17	3.43	.001	.06	.25
	T1 Girls	.01	.05	.01	.15	.878	-.10	.12
	T1 Disability	.08	.06	.06	1.33	.182	-.04	.21
	T1 Low-Q SES household	-.12	.10	-.05	-1.24	.214	-.33	.07
	T1 High-Q SES household	-.05	.06	-.04	-.92	.358	-.17	.06
	T2 Social acceptance competence	.23	.05	.21	4.19	< .001	.12	.34
	T2 Physical appearance competence	.14	.04	.15	2.99	.003	.04	.23
	T2 Low-Q cope solve the problem	-.12	.07	-.09	-1.78	.075	-.27	.01
	T2 Non-productive coping	-.01	.01	-.13	-2.71	.007	-.02	-.01
	T2 Effort motivation	.07	.01	.33	6.75	< .001	.05	.09
	R^2^ Change = .293, R = .728, R^2^ = .530, adjusted R^2^ = .511, F change for R^2^ = .31.320, *p* > .05							
	*F*_[10, 251]_ = 28.259, *p <* .001							
**Step 4: T1 School belongingness scores + Covariates+ Student personal factors**+ **family factors+ school and classroom factors**	(Constant)	1.64	.35		4.61	< .001	.94	2.34
	T1 School belongingness	.13	.04	.15	3.14	.002	.05	.22
	T1 Girls	.01	.05	.01	.32	.746	-.09	.12
	T1 Disability	.06	.06	.04	1.07	.283	-.05	.18
	T1 Low-Q SES household	-.15	.09	-.06	-1.54	.124	-.34	.04
	T1 High-Q SES household	-.11	.05	-.07	-1.79	.073	-.22	.01
	T2 Social acceptance competence	.20	.05	.18	3.82	< .001	.09	.30
	T2 Physical appearance competence	.11	.04	.12	2.59	.010	.02	.20
	T2 Low-Q cope solve the problem	-.14	.06	-.09	-2.07	.039	-.27	-.01
	T2 Non-productive coping	-.01	.01	-.14	-3.21	.001	-.02	-.01
	T2 Effort motivation	.04	.01	.20	3.90	< .001	.02	.06
	T2 Low-Q task goal orientation	-.18	.06	-.14	-3.00	.003	-.31	-.06
	T2 High-Q task goal orientation	.31	.09	.15	3.39	< .001	.13	.50
	T2 Tolerance to disability	.06	.01	.15	3.52	< .001	.02	.09
	R^2^ Change = .064, R = .771, R^2^ = .594, adjusted R^2^ = .573, F change for R^2^ = 13.102, *p <* .001							
	*F* [13, 248] = 27.906, *p <* .001							

#### T1 Primary school belongingness score

Pre-transition belongingness was found to have a significant positive association with secondary school belongingness at T2 (Beta = .15, *p =* .002).

#### Student personal factors

Social acceptance (Beta = .18, *p <* .001) and physical appearance competence (Beta = .12, *p =* .010) continued to be assets, while use of non-productive coping strategies (Beta = -.14, *p <* .001) and low-Q level problem-solving coping strategies (Beta = -.09, *p* = .039) were each significant risks to secondary school belongingness. The positive association between pursuing effort-goal motivational orientations on school belongingness was unique to secondary school (Beta = .20, *p <* .001).

#### School and classroom factors

The task-goal orientation of secondary school classrooms was a significant factor in determining school belongingness. Those who identified their year level classes to be low on task-goal orientations were less likely to belong (Beta = -.14, *p =* .003). The positive association between perceiving one’s teachers to frequently endorse task-goal structure and school belongingness was unique to secondary school (Beta = .15, *p <* .001). Positive associations between tolerance to disability and school belongingness were also identified at T2 (Beta = .15, *p <* .001). Identifying one’s class as highly autonomous did not positively contribute to secondary school belongingness. No differences in secondary school belongingness due to gender, health status and SES-level were identified. Family factors were not associated with school belongingness at T2.

## Discussion

This study intended to bridge the gap in the literature on school belongingness across the primary-secondary school transition, and outline the most influential personal student and contextual factors associated with belongingness. Analyses revealed school belongingness was stable across the primary-secondary school transition for the students in our study. No within-group variability in school belongingness due to gender, disability or household-SES was found. Students who reported higher belongingness in primary school were more likely to report higher belongingness in secondary school. Unique to the secondary-school belongingness model was the influence of student effort-goal orientation and perception of their school’s tolerance to disability on their belongingness scores. Several student personal factors (i.e., competence, coping skills) and school factors (i.e., low-level classroom task-goal orientation) that have previously been found to influence belongingness in primary school [46], continued to influence belongingness in secondary school; even after their prior belongingness scores were controlled. The findings of this study highlight the importance for primary schools to promote and assess school belongingness among students at an early age, and provide secondary schools with an overview of students’ belongingness profiles as part of the hand-over documentation in the transition from primary to secondary school. Our findings also highlight the need for secondary schools to organise classrooms goals, tasks and assignments, and foster pluralism among all students in such a way as to promote school belongingness.

Student personal attributes such as social competence, physical appearance competence and coping skills were significantly associated with belongingness in secondary school, even after primary school belongingness scores were considered. The study’s results substantiate prior literature on the influence of peer affiliation [90, 91], physical appearance competence [92–94], and coping skills on student adjustment in school [95, 96]. These findings emphasise the ongoing need for both primary and secondary schools to continue delivering life-skills training (around social skills, coping skills and optimism) to foster school belongingness in all their students, irrespective of disability, gender or household-SES [97, 98]. Based on these results, there is a need for programs that assist students to analyse and deconstruct body image ideals and media messages, placing emphasis on teaching the value of personal character and individual strengths over physicality [99]. With regards to coping skills, the current study’s results bring to attention the need for schools to afford students with opportunities to problem-solve when faced with a variety of challenges within and outside of school. In addition, support should be provided to those who choose non-productive strategies to deal with life stressors (such as worrying, ignoring the problem at hand and self-blame), as these students are at risk of reporting lower feelings of belongingness in both primary and secondary school.

When considered in conjunction with the primary school belongingness model [46], the findings of the current study suggest that at different times of the primary-to-secondary school transition, school belongingness is influenced by different motivational goals adopted by students. In contrast to previous results showing that primary school belongingness is associated with social-goal orientations [46]; the current study found that students who pursued effort-goals in secondary school were more likely to feel they belonged. This finding suggests that implementation of an effort-goal motivational culture in secondary schools that focuses on students’ strength and the process of learning can enhance their school belongingness. This may be especially important for students with disabilities because repeated failures to perform at normative levels can result in reduced effort goal orientation [100] and belongingness.

The moderate association found between students’ belongingness scores across the transition, together with the absence of any significant reduction in scores, suggest that students who enter secondary school with lower belongingness continue to be disadvantaged over time. Given that prior research reports that the perception of school belongingness decreases as students’ progress through the secondary years of school [16, 101, 102], the findings of the current study are encouraging, suggesting that Australian students’ perception of school belongingness is stable across the primary-secondary school transition.

School and classroom factors explained just over 6% of the variability in secondary school belongingness, after prior belongingness scores, demographic and personal attributes of the individual student were controlled. Classroom task-goal structure and tolerance to disability were the two most significant contributors. A non-linear relationship between classroom task-goal orientation and school belongingness was found. Students who reported their classrooms to have high-level task-goal structure were more likely to belong; while those who felt their classrooms had low task-goal structure reported lower belongingness. Achievement goal theorists would argue that task-oriented settings reduce the feeling of being controlled by the teacher and help establish consistency and clarity of goal expectations amongst students [103–105]. Thus, a student’s realisation that he or she is capable of self-regulating in a new school setting could nurture a feeling of belongingness and membership to that school [104]. In the current study, task-goal orientation of classrooms was measured in terms of the instructional style, assignments, and degree of competitiveness afforded to students in class. This finding stresses the importance for secondary school teachers to organise classrooms goals, tasks and assignments in such a way as to facilitate students’ belongingness to the classroom and wider school environment.

Support for, and sensitivity to, student diversity is an important dimension of the social climate of educational settings that impacts on student adjustment [82, 106, 107]; a premise that is validated by our study’s findings. Students who perceived their secondary school classrooms to be more accepting of individual differences due to disability were more likely to report higher belongingness. Although students perceived their schools to be equally tolerant to disability across transition (as evidenced statistically by the stable score); only in secondary school did this factor influence the outcome. This suggests that information given in early primary school about impairments, disabilities and everyday consequences may promote understanding and acceptance of diversity within the classroom, perhaps becoming beneficial in later years of schooling. According to the theory of attribution, an individual’s reaction to others is related to their understanding of responsibility [108].Therefore, education aimed at explaining differences as a consequence of disability, and ‘classmate responsibility’ in terms of the influence an individual’s behaviour has on others, can inform intervention promoting the acceptance of disability [109].

An unexpected finding was that involvement in academic classroom activities did not influence belongingness in secondary school, and may be attributed to a reduction in average levels of classroom involvement scores of the sample, or alternatively, may have been a function of how the involvement score was measured. Classroom involvement as a construct was operationalised in our study in terms of discussing ideas in class and explaining how to solve problems. This means that in secondary school, being involved in academic classroom activities does not influence school belongingness in the same way it does in primary school. Similarly, unlike the primary school belongingness model [46], belonging to an autonomy-granting and culturally pluralistic classroom did not influence belongingness in secondary school.

Associations between being bullied at school and reduced school belongingness are well established in the literature [110, 111]. In the current study, students reported less bullying in secondary school. This trend of reduced bullying six months after the transition to a new setting could be attributed to several factors, such as: the timing of data collection (i.e., post-transition data were collected six months after transition into secondary school, after peer hierarchies were established and students were beginning to fit into peer groups); the transition trend displayed in the study (i.e., the shift from the public/government education to the privatise/non-government education sector); or the use of a single item to assess bullying that might have precluded the relative importance of component of bullying using physical, verbal, social and electronic modes. Given existing evidence on the detrimental effects of bullying on social and emotional health of students [112], our findings of the lack of any significant contribution of being bullied in secondary school on concurrent school belongingness (once prior belongingness scores were considered) is encouraging. Further longitudinal research into this area is warranted to better understand and validate our findings.

Structural attributes of the school setting, such as household-SES, sector, and organizational model of schooling, did not contribute towards the school belongingness model before and after the transition [113]. This finding is contrary to that found in studies from the U.S. [114, 115]. It suggests that in the case of our Australian cohort, classroom attributes agreeable to change have a more dominant influence on school belongingness than fixed structural attributes of the school, which are often resistant to change. This is encouraging in light of growing assertions that the trend to enrol students in private schools in Australia may be exacerbating student separation by level of household-SES [113].

In summary, the current study makes a significant contribution to the literature on school belongingness across the primary-secondary transition. It presents:

1. The significant personal student and school factors associated with belongingness in secondary school students;
2. Evidence that students’ belongingness scores remain stable across the primary-secondary school transition. No within-group variability in school belongingness change due to disability, gender and household-SES exist. Those who feel they belong in the final year of primary school are more likely to feel belonging in secondary school;
3. Evidence that personal student attributes such as coping skills, social acceptance, physical appearance competence and effort motivational goal orientations account for 90% of the variability in secondary school belongingness; followed by classroom maintenance attributes such as task-goal orientation and pluralism to disability;
4. Evidence that organisational and physical attributes of the school do not influence school belongingness, both in primary and secondary school, once background and personal student factors are considered; and
5. Evidence that in secondary school, family factors do not influence school belongingness, once personal student attributes and classroom factors are considered.

## Limitations

Our study has a number of limitations that can be accessed from our previous publications [45, 46]. To summarise a few, the study sample was restricted to metropolitan Perth and other major urban centres across WA. The study did not involve students from other regional and remote populations or other major metropolitan cities in Australia, which limits the generalisability of the findings. Second, we restricted inclusion into the health condition sub-group to those reported to have a disability or chronic illness and were enrolled in a regular class for at least 80% of the school hours per week; this limits the model’s generalisability to other school settings. In addition, the study had an attrition of 33% that could bias the results.

From a methodological point of view, it should be remembered that other models with other predictors may be as plausible as the ones presented. We did not explore how students with a disability conceptualised belongingness in school, and whether their perception differed from their typically developing peers. Qualitative inquiries could be beneficial to shed some light in this direction.

## Conclusions

Findings of the present study offer an empirical foundation for the need for school-based initiatives aimed at increasing belongingness in secondary school. The literature suggests that among youth in transition, those who are able to develop a better sense of belonging in school are more likely to have better outcomes, both in school and beyond [14, 116–118]. Further research is needed to example the interrelationship between factors outlined in the current study and academic, psychological and participatory outcomes in adolescents. Subgroup analyses would be beneficial to determine whether differences in belongingness due to type of disability exist. Inductive research using qualitative methods of enquiry could be beneficial to deepen our understanding of how students conceptualise school belongingness. Future research is needed to replicate the current study’s findings in a larger and more diverse sample to inform the development of policies and programs to promote school belongingness among primary and secondary school students.
